# Molecular basis for the fold organization and sarcomeric targeting of the muscle atrogin MuRF1

**DOI:** 10.1098/rsob.130172

**Published:** 2014-03-26

**Authors:** Barbara Franke, Alexander Gasch, Dayté Rodriguez, Mohamed Chami, Muzamil M. Khan, Rüdiger Rudolf, Jaclyn Bibby, Akira Hanashima, Julijus Bogomolovas, Eleonore von Castelmur, Daniel J. Rigden, Isabel Uson, Siegfried Labeit, Olga Mayans

**Affiliations:** 1 Institute of Integrative Biology, University of Liverpool, Biosciences Building, Crown Street, Liverpool L69 7ZB, UK; 2 Institut für Anästhesiologie und Operative Intensivmedizin, Universitätsklinikum Mannheim, Mannheim 68167, Germany; 3 Instituto de Biología Molecular de Barcelona, Barcelona Science Park, Barcelona, Spain; 4 Center for Cellular Imaging and Nanoanalytics (C-CINA), Biozentrum, University of Basel, Basel, Switzerland; 5 Institut für Toxikologie und Genetik, Karlsruhe Institute of Technology, Eggenstein-Leopoldshafen 76344, Germany; 6 Institut für Zell- und Molekularbiologie, University of Applied Sciences Mannheim, Mannheim 68163, Germany

**Keywords:** RBCC/TRIM fold, coiled-coil, COS-box, X-ray crystallography, electron microscopy, *ab initio* modelling

## Abstract

MuRF1 is an E3 ubiquitin ligase central to muscle catabolism. It belongs to the TRIM protein family characterized by a tripartite fold of RING, B-box and coiled-coil (CC) motifs, followed by variable C-terminal domains. The CC motif is hypothesized to be responsible for domain organization in the fold as well as for high-order assembly into functional entities. But data on CC from this family that can clarify the structural significance of this motif are scarce. We have characterized the helical region from MuRF1 and show that, contrary to expectations, its CC domain assembles unproductively, being the B2- and COS-boxes in the fold (respectively flanking the CC) that promote a native quaternary structure. In particular, the C-terminal COS-box seemingly forms an α-hairpin that packs against the CC, influencing its dimerization. This shows that a C-terminal variable domain can be tightly integrated within the conserved TRIM fold to modulate its structure and function. Furthermore, data from transfected muscle show that in MuRF1 the COS-box mediates the *in vivo* targeting of sarcoskeletal structures and points to the pharmacological relevance of the COS domain for treating MuRF1-mediated muscle atrophy.

## 2. Background

The regulation of protein catabolism by the proteasome system receives ever-increasing attention owing to its impact on the (patho)physiology of the eukaryotic cell. The TRIM protein family consists of E2 and E3 ubiquitylating proteins that arbitrate cellular processes, such as growth and differentiation, transcription, apoptosis and viral response [1,2]. Consequently, TRIMs have been linked to multiple pathologies, including cancer, familiar Mediterranean fever, Opitz/BBB syndrome, mulibrey nanism, thyroid carcinomas and myopathy [3–6]. Despite their functional diversity, TRIM proteins invariably share a tripartite fold consisting of a RING finger (R), one or two RING finger-like B-box domains (B) and a helical segment predicted to form a coiled-coil (CC) motif. This RBCC fold constitutes the constant N-terminal fraction of TRIM proteins, but variable domains can be found in C-terminal position (e.g*.* PHD, COS-box, PRY-SPRY). Those specific domains define the classification of the more than 70 members of the TRIM family into nine distinct classes (CI-CIX, where C signifies C-terminal subgroup) [7,8].

The function of the TRIM/RBCC fold is to serve as a scaffold that induces homo- and heteromeric interactions across diverse E2–E3 ubiquitylation systems, leading to their formation of pleiotropic complexes in the cell [2,9,10]. The CC domain is thought to be central to this function by contributing to position domains within the TRIM fold, promoting high-order assembly and mediating molecular targeting. However, CC domains from TRIMs have atypical sequences with poorly defined heptad-repeat compositions that confer on them complex associative properties and a pronounced tendency to aggregate. This hinders their characterization at the molecular level and, as a result, little understanding exists of their self-assembly process.

**Figure 1.**
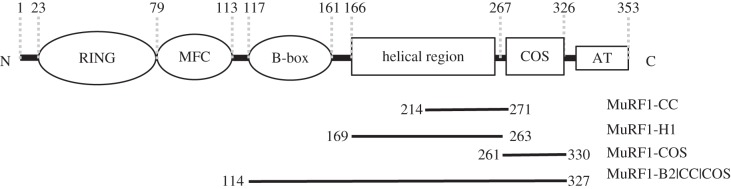
Schematic domain composition of MuRF1. MFC refers to an MuRF family specific motif, and AT denotes a C-terminal acidic tail, which is predicted to be unstructured. Constructs used in this study are indicated.

Muscle-specific RING fingers proteins (MuRFs) are E3 ubiquitin ligases that associate with the sarcomeric cytoskeleton reportedly through their CC domains [11]. MuRFs form the C-II TRIM class [7], whose C-terminal specific fraction contains a COS (C-terminal subgroup One Signature)-box motif and an intrinsically disordered acidic tail (figure 1). There are three known members of the MuRF family—MuRF1 [12], MuRF2 [13] and MuRF3 [14]—all involved in controlling the trophicity of striated muscle tissue. The three MuRFs are encoded by different genes but are remarkably conserved: approximately 81% sequence identity across their RB fractions and approximately 36% in their CC domains [6]. MuRF1 is the best-studied member of the family. It is strongly upregulated by atrophic stimuli, such as immobilization, denervation, nutritional deprivation, ageing and disease (e.g. cancer, sepsis and renal failure) [15–17]. MuRF1 targets components of the contractile sarcoskeleton; namely, myosin [18,19], troponin-T and titin [12,20,21]. Thus, it is regarded as the critical E3 ligase that acts on the cytoskeleton *in situ*, contributing to the disassembly of the myofibril. MuRF1 also appears to have signalling roles in the cell as it interacts with a broad range of cellular factors, including ubiquitin carboxyl-terminal hydrolase 13 (USP13), the SUMO E2 ligase Ubc9 and the transcription regulator GMEB-1 [22]. MuRF1 deletion attenuates muscle wasting and it is a pursued pharmacological target [15,23]. Despite this, the structural and functional differences between MuRFs are poorly understood, as is the balance of their expression across muscle types and during development [24,25]. MuRF2 and MuRF3 are not transcriptionally upregulated by atrophic stimuli, but they act synergistically with MuRF1 (e.g. myosin is co-degraded by MuRF1 and MuRF3 [26], while MuRF1 and MuRF2 jointly modulate cardiac hypertrophy by acting on CARP/EEF1G [21]). Such functional coupling might reflect the formation of MuRF hetero-oligomers in the cell, as is characteristic of TRIM proteins.

The molecular understanding of MuRF1 targeting is scarce, with only the interaction with the titin myofilament having been studied *in vitro* [11,12]. Using recombinant samples, we showed that MuRF1 binds M-line titin with high affinity through its helical domain (HD) [11]. The binding site in titin is formed by a tandem of Ig-Ig-Fn domains (A168-A170) just N-terminal to titin kinase. We previously characterized this tandem structurally at atomic level and identified the determinants of its binding [11]. By contrast, little is known about the structure of MuRF1 and its CC scaffold, which is central to molecular targeting. Here, we study the full-length HD of MuRF1, spanning its atypical CC sequence and the COS-box flanking domain. Our data reveal the interrelation of these motifs both structurally and functionally within the TRIM fold of MuRF1, and, in particular, the high significance of the COS-box in sarcomere targeting.

## 3. Results

### 3.1. The helical domain of MuRF1 has low propensity to coiled-coil formation

**Figure 2.**
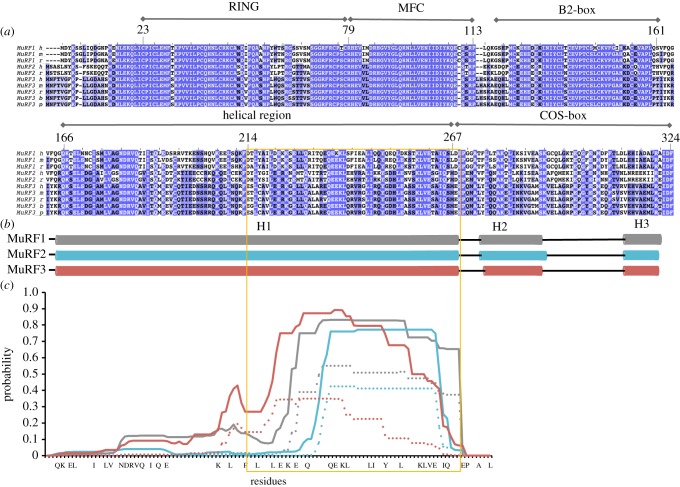
Sequence analysis of the HD of MuRFs. (*a*) Sequence alignment of known MuRF sequences (MuRF1, MuRF2, MuRF3; h = human, m = mouse, r = rat, b = bovine, p = orangutan). The unstructured acidic tail is excluded. The colour code reflects sequence conservation as identity percentile: dark blue > 80%, light blue > 60%, light grey > 40%, white ≤ 40%. (*b*) Secondary structure prediction of the HD fraction (lower panel in (*a*)) of human MuRF1 (grey), MuRF2 (cyan) and MuRF3 (red). Cylinders indicate helices. (*c*) Prediction of CC regions in human MuRFs (colour code as above). The total probability for CC formation is shown as a solid line, with the probability for dimeric CC assembly indicated by a dashed line. Boxed in yellow is the sequence identified here as the CC-compatible segment of MuRF proteins. Strictly conserved residues are shown in the *x*-axis.

To estimate the associative properties of the HDs of MuRFs, we predicted their potential for CC formation. CC motifs consist of two to five amphipathic α-helices that wind around each other to form, typically, a left-handed supercoil, thereby inducing protein oligomerization [27]. CC sequences are characterized by a heptad-repeat of amino acids denoted by *a* to *g*. Positions *a* and *d* classically harbour hydrophobic residues, which constitute the structural core of the motif. Positions *e* and *g* often host charged groups that form intra- and inter-helical salt bridges crucial to fold stability and interchain registry. An initial prediction of secondary structure content indicated that the helical region of MuRFs consists of a long, uninterrupted helix (helix H1), followed by two short C-terminal helices (helix H2 and H3) linked by loops and mapping to the COS-box motif (figure 2). This prediction is consistent with a previous study that estimated the secondary structure content of the helical region of MuRF1 to be 70% α-helix and 30% random coil, based on circular dichroism data [11]. Predictors indicated that only the C-terminal end of the long helix H1 is compatible with CC formation. The prediction was consistent for all three MuRFs, although MuRF2 had the shortest predicted CC-segment owing to the presence of bulky residues (M227 and F266) in core heptad positions *a* and *d*. Such groups are poorly accommodated in the limited interface of coiling α-helices [28]. The tendency for CC formation was modest for all MuRFs, as reflected by the low probability scores (figure 2*c*). The scores, in addition, could not resolve a preference for dimeric or trimeric association as both states yielded comparable probability values. However, previous studies that used size exclusion chromatography coupled to multi-angle laser light scattering (SEC-MALLS) on the helical fraction of MuRF1 showed it to form dimers [11,29]. In the light of these data, the predictions led us to anticipate that the CC-prone, C-terminal end of helix H1 must form dimeric CC motifs, thereby being a molecular determinant of self-assembly in MuRFs.

### 3.2. Crystal structure of the MuRF1 CC segment reveals a tetrameric palindrome

**Figure 3.**
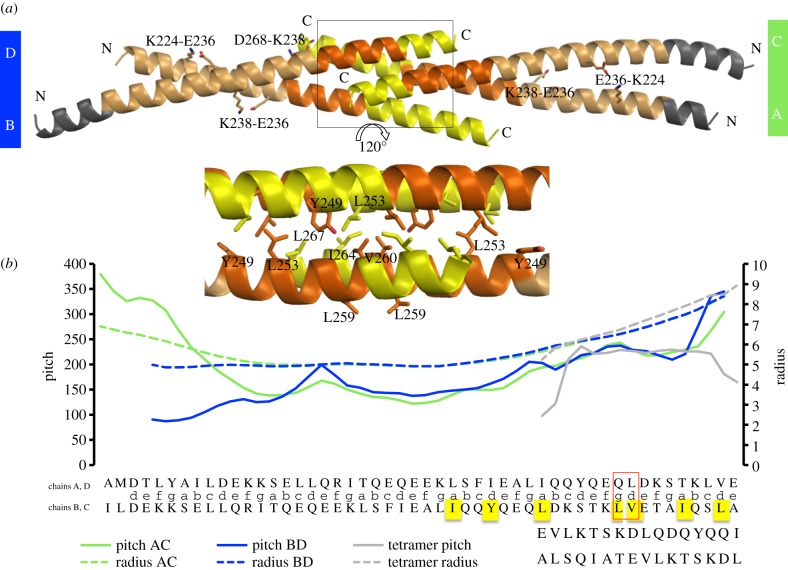
(*a*) Crystal structure of MuRF1^CC^, where helices have been coloured to indicate: no interchain assembly (grey), parallel interaction (beige), antiparallel interaction (yellow), simultaneous parallel and antiparallel interaction (orange). Salt bridges are labelled. The inset (central panel) shows the hydrophobic core formation of the tetrameric region. (*b*) Twister analysis of each MuRF1^CC^ half. The structural point to which the values correspond is indicated by the sequence (in dimeric or tetrameric state) displayed in the *x*-axis. Solid lines indicate pitch and dashed lines radius. The pitch and radius vary along the length of the molecule, but the values of the central region of each dimeric half are close to those of a canonical dimeric CC, where pitch approximates 150 Å and radius 5 Å [30]. The analysis of knobs-into-hole packing of each dimer using SOCKET [29] indicated that 18 residues in the AC dimer exhibited a conventional CC packing (7,7,4 repeat), while the BD dimer had 25 residues in CC arrangement (7,7,7,4 repeat) (electronic supplementary material, figure S1). In the sequence that follows from this, TWISTER shows the opening of the coil and a resulting helical phase transition (between residues 252-QL-253 in chains A and D, respectively, partnered to chains C and B at motif 259-LV-260; highlighted by a red box) that defines the switch from a dimeric to a tetrameric association.

To gain an insight into the assembly of the CC-prone segment identified, we elucidated the crystal structure of the corresponding fragment from human MuRF1 (MuRF1^CC^) to 2.1 Å resolution (table 1). The crystals contained four copies of the MuRF1^CC^ chain in their asymmetric unit. These assembled into two parallel dimers, each having an ‘open scissor’ conformation that intercalated through their C-terminal ends to form a palindromic, inverted tetramer (figure 3). This packing resembles that of the CC domains from the postsynaptic density protein Homer (PDB code 3CVE), Ndel1 (2V66) and BST2/tetherin (3MKX), although sequence similarity between these and MuRF1^CC^ is not detectable. In MuRF1, however, the chains within each dimer are out of registry by one full heptad-repeat and their crossing angle is broad, leading to the unpairing of both N- and C-termini. The resulting exposure of hydrophobic residues at the C-terminus permits the formation of an interchain hydrophobic core that supports the assembly of the inverted tetramer (inset in figure 3). Other than that core, the contacts within each dimer are few, with only two salt bridges (K224-E236’ and E236-K238’) providing chain recognition (analysis of chain interfaces used PISA [31]). One additional salt bridge (K238-D268’’; chains DA in figure 3*a*) is present in the structure, but it contributes to tetramer stabilization.

**Table 1.** Diffraction data statistics and model refinement parameters.

space group	P2_1_
unit cell dimensions	*a* = 70.79 Å, *b* = 24.41 Å, *c* = 75.39 Å, *β* = 107.65°
X-ray data	
beamline	I03 (diamond)
detector	ADSC Q315r
wavelength (Å)	0.97
resolution (Å)	20.00–2.10 (2.15–2.10)
no. unique reflections	14 626 (1008)
Rsym (I)	2.7 (45.0)
multiplicity	3.64 (3.74)
completeness (%)	97.7 (99.3)
I/*σ* (I)	17.24 (3.31)
model refinement	
no. reflections in working/free set	13 899/725
no. protein residues	228^a^
no. solvent molecules/buffer molecules	55/20^b^
R-factor/R-free (%)	21.18/26.15
RMSD bond length (Å)/bond angle (°)	0.006/0.836
Ramachandran statistics	
favoured/allowed/outliers (%)	99.07/0.46/0.47

^a^Out of a total of 244 amino acids, 16 residues were structurally disordered and are missing from the model (corresponding to 6.5% of the structure). The missing residues are as follows: chain A (G271), chain C (G-3, E269, P270, G271), chain B (E269, P270, G271), chain D (G-3, A-2, M-1, D214, D268, E269, P270, G271).

^b^Ordered buffer components are glycerol and acetic acid.

A further analysis with TWISTER [32] (figure 3*b*) and SOCKET [28] (electronic supplementary material, figure S1) was used to identify stretches in each chain that formed parallel, antiparallel or simultaneous parallel/antiparallel interactions. The analysis revealed that MuRF1^CC^ can establish manifold interactions, being able to support concurrently the formation of parallel and antiparallel dimers and tetrameric helical bundles. These promiscuous self-associative interactions are in agreement with the mixed probability scores of the CC predictions. This led us to question the prevailing view that this MuRF1 region associates into robust CC motifs and that it directs the productive quaternary assembly of MuRF1.

### 3.3. Coiled-coil fraction of MuRF1 does not assemble productively in solution

Given the unexpected assembly of MuRF1^CC^ in the crystalline state, we studied its association in solution using SEC-MALLS. This technique yields an accurate determination of molecular mass (MM) without being influenced by molecular shape, an important consideration when dealing with strongly anisometric molecules. SEC-MALLS measurements yielded an average MM of 11.8 kDa (table 2; electronic supplementary material, figure S3*a*). This value is intermediate between the calculated MM of a monomer (7 kDa) and a dimer (14 kDa) of this sample. This indicates that the tetrameric crystalline state does not predominate in solution, where the sample appears to form mostly dimers, probably in equilibrium with a monomeric fraction.

**Table 2.** SEC-MALLS measurements. MM calculated from sequence data (MM_calc_) is quoted for the monomeric chain, with the value for a dimer given in brackets. Experimental MM values (MM_exp_) have been measured using SEC-MALLS (data shown in the electronic supplementary material, figure S3).

	CC^214–271^	H1^169–263^	B2|CC|COS^114–327^	^a^HD^166–327^
MM_calc_ (kDa)	7 (14)	11.3 (22.6)	24 (48.1)	17.4 (34.7)
MM_exp_ (kDa)	11.8	37.4	49.2	32.3

^a^Given here for comparison, values reported in [28] for the full-length HD of MuRF1 spanning H1 and COS regions.

To investigate whether the extraction of MuRF1^CC^ from its molecular context could have weakened its self-association, we assayed next an MuRF1 construct spanning the full-length of helix H1 (MuRF1^H1^; figure 1), which probably constitutes the entire CC motif. The expectation was that this sample would show a stable, canonical, dimeric association. However, SEC-MALLS data revealed that the sample is trimeric and/or tetrameric in solution (table 2; electronic supplementary material, figure S3*b*). The assemblies in solution may reproduce the interactions observed in the crystal structure of MuRF1^CC^, but these might now be stabilized by the longer length of the interacting chain. It can be concluded that the long helix H1 of MuRF1 does not assemble into native CC dimers.

### 3.4. Coiled-coil flanking motifs modulate the formation of MuRF1 rod-shaped dimers

The irregularities in MuRF1^H1^ association led us to investigate the role of CC flanking motifs in assembly. Using SEC-MALLS, we analysed the oligomeric state of an MuRF1 construct comprising the full-length helix H1, the preceding B2 box and the subsequent COS-box (MuRF1^B2|CC|COS^; figure 1). The data **(**table 2; electronic supplementary material, figure S3*c*) showed that the sample forms a range of oligomeric species, including high-order aggregates, but that a sizeable fraction forms small assemblies with an average MM of 49.2 kDa. This value is in excellent agreement with the theoretical MM of 48.1 kDa for a dimer of this construct, confirming that the dimer (and not the tetramer) is the ground association state of this sample.

**Figure 4.**
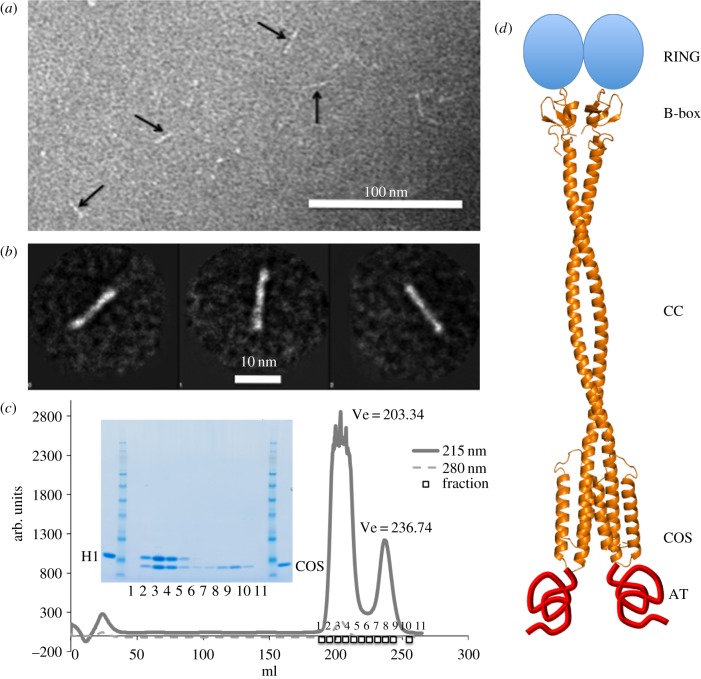
Characterization of MuRF1^B2|CC|COS^. (*a*) Electron micrograph of negatively stained MuRF1^B2|CC|COS^ samples corresponding to the outermost tail fractions of size-exclusion chromatograms containing dimeric assemblies (electronic supplementary material, figure S3). (*b*) Gallery of three major class averages obtained by using the processing software EMAN1. (*c*) Complexation of MuRF1^H1^ and MuRF1^COS^ samples monitored by size-exclusion chromatography. The complex was formed by mixing the samples in a molar ratio of 1 : 2.5 in 20 mM Tris–HCl pH 7.5, 200 mM NaCl, 1 mM DTT followed by incubation for 1 h at 4°C. The mixture was run on a Superdex 200 HiLoad 26/60 column. Chromatogram and associated SDS-PAGE are shown. MW marker is SeeBlue Plus2 Pre-stained standard (Invitrogen) (samples are proximal to the 6 kDa band). (*d*) Proposed quaternary structure of MuRF1 compiling known and predicted structural information on MuRF1. The structure of the B2-box dimer is that previously elucidated by X-ray crystallography (PDB 3DDT) [29]; the model for the HD and its dimeric assembly is as deducted in the current study.

To explore the global conformational features of MuRF1^B2|CC|COS^, we imaged the dimeric population fraction using electron microscopy on negatively stained samples (figure 4*a,b*). Micrographs showed a rod-like morphology of approximately 17 ± 3 nm length and 2.6 ± 0.36 nm cross-section (*n* = 614). This overall shape suggested that, as expected, the HD forms an elongated shaft with the B2-box in apical position. However, the molecular length was shorter than anticipated and could be explained by just the length of helix H1 (29 helical turns with a pitch of 5.4 Å approximates 15.6 nm) plus the B2-box (approx. 2 nm). This led us to infer that the two short C-terminal helices (i.e. the COS-box) must fold against the B2-CC fraction.

### 3.5. The C-terminal COS-box of MuRF1 is predicted to have a spectrin-like fold

Next, we explored the fold of the COS-box through *ab initio* modelling. This motif has no homology with any protein of known structure, preventing the application of comparative modelling. In *ab initio* modelling, the three-dimensional structure of proteins is derived from their amino acid sequence by stitching together suitable protein fragments using simulated annealing. The method is particularly successful when applied to all-α proteins [33] owing to the greater accuracy of their secondary structure prediction and the relatively limited modes of helical packing compared with the variable twists of β-sheets. Here, we employed the two leading *ab initio* modelling programs: Quark [34] and Rosetta [35]. Quark assembles fragments of variable length identified by fold recognition methods. Only available as a server, it returns a set of ten predictions and estimates of model reliability as TM-scores. Rosetta assembles fragments of 3- and 9-residue length identified using PSI-BLAST. At the fragment assembly stage, numerous models are clustered by structural similarity and centroid representatives of large top clusters considered as candidate fold predictions. The appearance of a large top cluster is generally indicative of accurate fold predictions.

**Figure 5.**
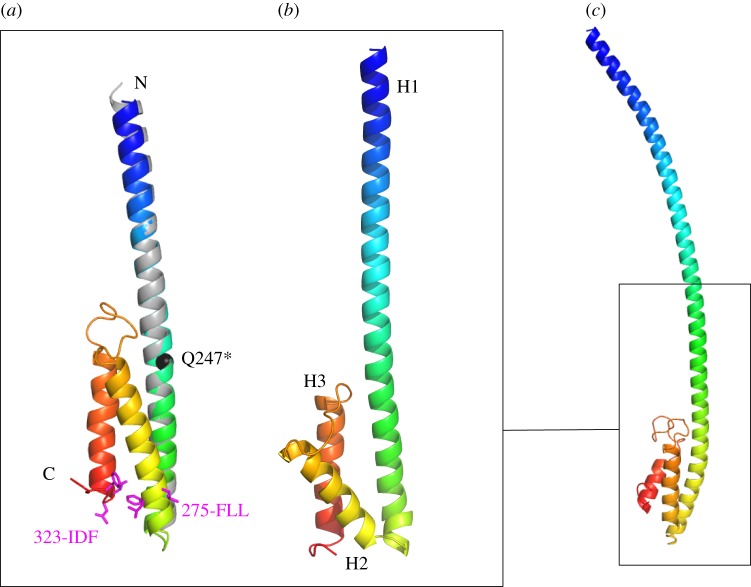
*Ab initio* modelling of MuRF1 COS-box. (*a*) Top Quark model spanning MuRF1^CC^ plus the subsequent COS-box region coloured in a blue-to-red gradient. The model is superimposed on the crystal structure of MuRF1^CC^ (grey). The pathogenic Q247* mutation is shown in black and motifs previously identified to mediate microtubule binding in protein MID1 are in magenta [7]. Additional Quark models are shown in the electronic supplementary material, figure S2. (*b*) Rosetta-calculated model of the same segment derived from a cluster of 150 models. A comparison of Quark and Rosetta models is shown in the electronic supplementary material, figure S2. (*c*) Top Quark model of the full-length HD of MuRF1 (the degree of bending of the long helix H1 is not meaningful).

First, we modelled the sequence spanning the crystallographic MuRF1^CC^ and the COS-box, as EM data suggested that the COS-box might require the preceding MuRF1^CC^ portion for packing. Models calculated using Quark (figure 5*a*) were in excellent agreement with the crystal structure in predicting MuRF1^CC^ as a long α-helical shaft. They displayed the C-terminal COS-box as a compact arrangement, where two short helices folded into an α-hairpin that packed against the shaft fraction. The resulting three-helix bundle resembled a minimal version of the spectrin fold, where two helices lie parallel to each other and the third is a cross-connector [36]. Nine out of the 10 predictions (electronic supplementary material, figure S2) returned by Quark shared this same broad fold and had predicted TM-scores of 0.51–0.45 (over the threshold of 0.3 that indicates statistical significance). *Ab initio* modelling in Rosetta using the standard protocol was unsuccessful, with the program overriding long helices suggested by secondary structure predictions to produce compact, globular folds. This is a known limitation of Rosetta when handling anisometric structures [33]. Thus, we next provided Rosetta with the crystal structure of MuRF1^CC^ as a fixed fraction. This resulted in the largest cluster containing 150 of the total 1000 models, indicative of a satisfactory result (figure 5*b*). The model shared the same topology with Quark models (electronic supplementary material, figure S2*d*), this consistency being a further indication of reliable modelling.

We then performed *ab initio* modelling in Quark of the full-length HD of MuRF1, comprising helix H1 and COS-box (MuRF1^HD^). The models (figure 5*c*) represented the N-terminal fraction of the domain as a single uninterrupted helix and were consistent with those of MuRF1^CC^ plus COS-box described above. This result is in excellent agreement with secondary structure predictions **(**figure 2**)** and EM data.

Interestingly, a comparison of *ab initio* models with the crystal structure of MuRF1^CC^ revealed a remarkable similarity in helix packing (electronic supplementary material, figure S4). In the MuRF1^CC^ tetramer, helices arrange themselves along the same interfaces as those occupied by the COS-box α-hairpin in the *ab initio* models. This suggested that the crystallographic arrangement was a compensatory conformation aimed to satisfy naturalistic interfaces.

Finally, we sought validation of the *ab initio* models by testing the interaction of independently produced samples of MuRF1^H1^ and the COS-box α-hairpin (MuRF1^COS^). Co-segregation of both samples in size-exclusion chromatography (figure 4*c*; electronic supplementary material, figure S5) confirmed the interaction of the two segments. Our earlier work [11,29] showed the HD of MuRF1 (spanning H1 and COS-box) to be dimeric, similar to MuRF1^B2|CC|COS^ in this work. However, individually MuRF^H1^ forms higher assemblies (table 2). This led us to conclude that the interaction of H1 and COS-box prevents the non-native association of the HD, and thus that the COS-box is required to achieve productive homodimerization.

### 3.6. *In vivo* expressed COS-box targets sarcomeric structures similar to full-length MuRF1

**Figure 6.**
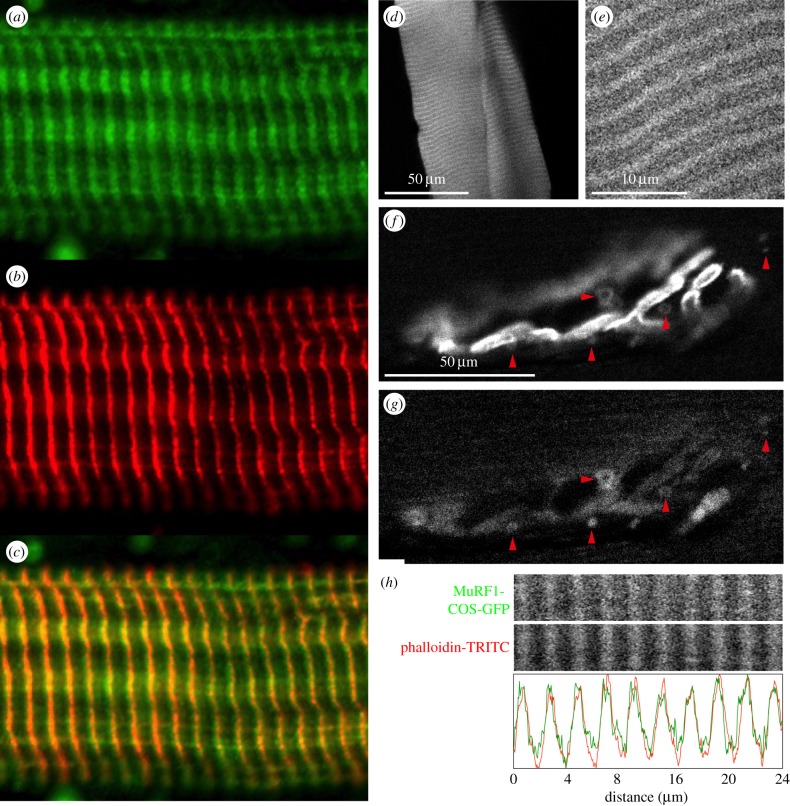
MuRF1 COS-box targeting of myocellular structures *in vivo*. (*a*–*c*) Localization of endogenous MuRF1 within sarcomeres. Muscle tissues were dissected from mice 5 days post-denervation, single myofibrils prepared from *M. gastrocnemius* and immunostained for (*a*) MuRF1 or (*b*) desmin. A merge of (*a*) and (*b*) indicates that endogenous MuRF1 under muscle stress targets predominantly (*c*) the Z-disc region and to a lesser extent the M-line. (*d–g*) *In vivo* targeting of transiently expressed MuRF1 COS-GFP fusion protein in transfected skeletal muscle. Depicted is (*d,e*) the enrichment of COS-GFP in sarcomeric striations as well as (*f*,*g*) an enrichment in puncta containing endocytic AChR ((*f*) α-bungarotoxin staining; (*g*) COS-GFP; arrowheads mark COS puncta that are also positive for AChR). A negative control corresponding to transfected GFP alone is shown in the electronic supplementary material, figure S6. (*h*) After fixation and staining with phalloidin-TRITC against f-actin, longitudinal sections of the muscles depicted in (*d*–*g*) show enrichment of COS-GFP in the Z-line/I-band, mimicking the distribution of endogenous MuRF1. Faint immunopositive signal on the M-line (*a*) is also present with COS-GFP *in vivo* (*e*) but does not stand fixation and staining (*h*).

We tested whether the COS-box contributes to sorting MuRF1 to its *in vivo* locations. For this, we first confirmed the localization of endogenous MuRF1 by immunostaining (figure 6*a–c*). This detected MuRF1 mostly in the Z-disc (consistent with its interaction with Z-disc proteins [20,21]) and also, more discretely, in the M-line region (consistent with its binding to titin A168–170 [11,12]) (figure 6*a–c*). Endogenous MuRF1 is also known to localize to the neuromuscular junction [37]. Then, we transfected the tibialis anterior (TA) muscle of adult mice with a COS-GFP fragment. *In vivo* imaging of the overexpressed COS-box showed a regular pattern of striations in the myofibril, indicative of its targeting of defined sarcomeric structures (figure 6*d,e*). In addition, GFP fluorescence was also present in punctate structures co-localizing with endocytic acetylcholine receptor at the neuromuscular junction (figure 6*f,g*). To determine the precise localization of COS-GFP in the sarcomeric striations, sections of the imaged muscles were prepared and stained against f-actin using the marker phalloidin-TRITC (figure 6*h*). This assigned the predominant *in vivo* targeting of COS-GFP to the Z-line/I-band region. The fainter binding at the M-line was no longer detectable, probably having been disrupted by the fixation procedure. On the whole, these data indicate that the localization of COS-GFP is consistent with that of endogenous MuRF1. Furthermore, the findings complement a recent study on a pathogenic mutation of MuRF1, Q247*, linked to hypertrophic cardiomyopathy [38]. The mutation results in a truncated protein that lacks the COS-box. Truncated MuRF1 remains diffuse in the cytoplasm, no longer targeting sarcomeric structures and with a near-total loss of ubiquitinating function. It can be concluded that the COS-box is an important mediator of MuRF1 interactions *in vivo*, and that it is necessary and sufficient for the recruitment of MuRF1 to the sarcomeric cytoskeleton.

## 4. Discussion

CC domains are thought to drive the organization of TRIM proteins into functional assemblies. However, our analysis of the CC from MuRF1 (MuRF1^CC^ and MuRF1^H1^) suggests that this domain does not govern molecular order in this TRIM but that flanking domains modulate its associative function. It is not rare that CC domains extracted from their protein context no longer assemble natively. For example, the coil 2 from lamin A forms parallel dimers in its intermediate filament context, but antiparallel dimers in isolation [39]. To compensate for the promiscuity of the CC fold, many CC-containing proteins have additional motifs that condition the self-assembly of these domains. An example is the dimeric dystrophia myotonica protein kinase (DMPK), whose C-terminal CC domain forms robust, but artefactual, trimers in isolation [40]. DMPK assembles into functional homodimers by means of an N-terminal association motif that dictates the subsequent interaction of the CC fraction [41]. In MuRF1, the B2- and COS-boxes flanking helix H1 act as terminal clamps that secure the correct self-assembly of the CC. Our previous structural characterization of the MuRF1 B2-box showed that it forms homodimers in solution with high affinity [29]. Studies on other TRIM proteins have confirmed that B-boxes also form homodimers in those cases [42]. Thus, our data explain the need for the B2-box to precede and pre-define the chain registry of the CC domain so as to initiate its productive assembly by vicinal confinement. This provides a rationale for the universal presence in the TRIM fold of the B2 box immediately N-terminal to the CC motif, forming an evolutionarily conserved core unit. Here, we also show that at the C-terminus, CC and COS-box sequences are integrated into a structural unit, together forming an HD, where the COS-box might prevent fraying of the CC and aid the formation of functional dimers. This is the first example of how a specific C-terminal domain is integrated within the conserved TRIM fold to modulate its structure and function. We anticipate that this architectural design is shared by all 10 TRIMs in classes I–III where the CC is immediately followed by a COS-box [5]. In addition to the close MuRF1 homologues MuRF2 and MuRF3, these classes include proteins such as the Opitz syndrome protein Midline-1 (MID1) and its homologue Midline-2 (MID2), the brain-specific TRIM9 that is seemingly linked to neuronal dysfunction in dementia, and Harprin (TRIM36), thought to regulate the acrosome reaction in sperm during fertilization.

We predict that the COS-box has a minimalistic spectrin-like fold. The spectrin fold has been particularly observed in microtubule-associated proteins [36], consistent with the role attributed to the COS-box [7,14]. The mutation of the conserved motifs FLQ and LDY in the COS-box of MID1 (respectively, 275-FLL-277 and 323-IDF-325 in MuRF1) were shown to independently abolish the interaction with microtubules [7]. *Ab initio* models in this study indicate that these motifs co-localize at the termini of the COS α-hairpin, at the base of the HD (figure 5*a*). This suggests that these motifs are important for the correct folding of the COS-box and/or that they form a key interaction locus in the fold. Furthermore, previous SPOT-blots identified a C-terminal sequence as the primary interaction site of MuRF1 with M-line titin [20]. The mapping of this sequence on our secondary structure predictions (figure 2) and on the *ab initio* models (figure 5) shows that it corresponds to helix H3. This helix is in the outermost position in the models and thus is largely accessible. We conclude that our model of MuRF1 COS-box rationalizes current binding data on this motif. Finally, we summarize the findings from this study in the proposal of an overall structural model of MuRF1 (figure 4*d*) that might guide its further functional study.

## 5. Conclusion

The change in demographics and inactive lifestyles is making muscle loss an endemic problem in the populations of developed countries. Hence, the study of pathways that regulate the degradation of muscle proteins is of high interest. Over 50 potential MuRF1 targets have been proposed based on yeast two hybrid screens, including sarcomeric proteins of the Z-disc and M-lines [20,21], and the neuromuscular junction [37]. Our findings suggest that the COS-box is a region of high structural and functional importance in MuRF1. It is a necessary interaction motif that mediates MuRF1 recruitment to myocellular structures, and thus the pharmacological perturbation of its targeting might open new avenues for the control of MuRF1-mediated atrophy of the myofibril.

## 6. Methods

### 6.1. Sequence analysis

Sequences of MuRF proteins were obtained from the UniprotKB database and aligned with Clustalw2 [43] using the BLOSUM matrix. Secondary structure predictions of the helical regions of MuRF1, -2, -3 used Jpred3 [44]. The probability for CC formation was calculated with MultiCoil [45] using a sequence window of 28 residues.

### 6.2. Cloning

Human MuRF1^CC^ (UniProtKB Q969Q1) was cloned into the vector pETM-11 (EMBL collection) using KpnI and NcoI restriction sites. This vector incorporates a His_6_-tag and a TEV protease cleavage site N-terminal to the target construct. MuRF1^H1^ (containing the mutation C173S) was cloned into pETM-20 (EMBL collection) using the restriction sites NcoI and BamHI, fusing an N-terminal His6-TRX tag to the target construct, cleavable by TEV protease. MuRF1^COS^ was cloned into the NcoI and Acc65I sites of pETZZ (http://babel.ucmp.umu.se/cpep/web_content/Pages/CPEP_09_vectors.html) to produce a TEV-protease-cleavable N-terminal His_6_-ZZtag construct. The expression clone of MuRF1^B2|CC|COS^ (containing the mutation C298S) has been previously reported [29].

The C-terminal fusion of the COS-box to GFP was obtained by cloning MuRF1^COS^ into the pEGFP-N1 vector using XhoI and EcoRI sites.

All constructs were verified by sequencing (Geneservice).

### 6.3. Protein production

MuRF1 samples were overexpressed in *Escherichia coli* Rosetta2 (DE3) or BL21 (DE3) (Novagen). Cultures were grown at 30°C up to an OD_600_ of 0.6 in Luria Bertani medium supplemented with 20 μg ml^−1^ chloramphenicol and 30 μg ml^−1^ kanamycin. Expression was induced by 0.75 mM isopropyl-β-d-thiogalactopyranoside (IPTG) and cultures were grown for a further 20 h at 20°C. Cells were harvested by centrifugation at 4°C. Bacterial pellet was resuspended in lysis buffer (50 mM Tris pH 8.0, 50 mM NaCl, 0.5 mM β-mercaptoethanol) containing a protease inhibitor cocktail (Roche). Lysis was carried out by French pressing in the presence of DNAse. The homogenate was clarified by centrifugation and the supernatant applied to a Ni^2+^-chelating His trap column (GE Healthcare) equilibrated in lysis buffer containing 20 mM imidazol. Elution used 200 mM imidazol. Tag removal was by incubation with TEV protease overnight at 4°C during dialysis against lysis buffer. The protein was then subjected to subtractive metal affinity chromatography followed by gel filtration on a Superdex 75 16/60 HL column or on a Superdex 200 26/60 HL column equilibrated in 20 mM Tris pH 7.5, 200 mM NaCl (GE Healthcare). The purified sample was stored at 4°C until further use. The production of MuRF1^B2|CC|COS^ was as reported [29].

### 6.4. Crystal structure elucidation

Crystals were grown at 20°C in hanging drops using 48-well plates (Hampton Research). Drops consisted of 1 µl protein solution at 14 mg ml^−1^ and 1 µl mother liquor containing 35% MPD, 0.1 M sodium acetate pH 4.5, 20% [v/v] glycerol and 20 mM NaF. Crystals grew as thin plates with dimensions of 400 × 100 μ^2^ in the measurable plane of the plate. For X-ray data collection crystals were flash frozen in liquid nitrogen. X-ray diffraction data were processed with XDS/XSCALE [46] (table 1). Crystals contained four MuRF1^CC^ chains in the asymmetric unit, corresponding to a solvent content of 46%. Analysis with POLARRFN revealed a twofold (*κ* = 180°) non-crystallographic axis contained within the crystallographic *ac* plane (*ω* = 90°, *ϕ* = 153°). Phases were obtained using ARCIMBOLDO [47]. Subsequent model building used COOT [48] and ARP/wARP [49], while refinement was in PHENIX [50]. Building of solvent and ordered buffer components used PHENIX and COOT.

### 6.5. Multi-angle laser light scattering

Measurements were performed on a Dionex BioLC HPLC connected to an 18-angle light-scattering detector and a differential refractometer (DAWN HELEOS-II and Optilab rEX, Wyatt). A Superdex 75 10/300 GL column (GE Healthcare) was used in 20 mM Tris pH 8.0, 50 mM NaCl at a flow rate of 0.75 ml min^−1^. Sample volumes of 1 ml were injected at a concentration of 1.5 mg ml^−1^. Samples eluting from the column passed through an in-line DAWN HELEOS-II laser photometer (*λ* = 658 nm) and an Optilab rEX refractometer with a QELS dynamic light-scattering attachment. Light-scattering intensity and eluent refractive index (concentration) were analysed using ASTRA v. 5.3.4.13 software to give a weight-averaged MM. To determine the detector delay volumes and normalization coefficients for the MALLS detector, a BSA sample (Sigma A-8531) was used as reference.

The SEC-MALLS analysis of MuRF1^B2|CC|COS^ samples was carried out as above but used a Superdex 200 10/300 prep-grade column equilibrated in 20 mM Tris pH 8.0, 100 mM NaCl. MuRF1^B2|CC|COS^ was injected at 0.7 mg ml^−1^.

### 6.6. Transmission electron microscopy and image processing

Aliquots of 5 μl sample were adsorbed onto a glow-discharged carbon film-coated copper grid, washed with three droplets of pure water and subsequently stained with 2% uranyl-acetate. Images were recorded using a Philips CM10 TEM (The Netherlands) operating at 80 kV on a Veleta 4 k CCD camera (Olympus, Germany).

Reference-free alignment was performed on manually selected particles from electron micrographs using the EMAN image-processing package [51]. Next, particle projections were classified by multi-variant statistical analysis. The class averages with the best signal-to-noise ratio were selected and gathered in a gallery.

### 6.7. *Ab initio* modelling

The MuRF1 sequence corresponding to residues 214–327 was submitted to the Quark *ab initio* modelling server [34]. For comparison, fragment assembly-based *ab initio* modelling was done with Rosetta using default parameters (*ab initio* protocol) to produce 1000 models [35]. This was done both with and without specifying that residues 214–271 must adopt the experimentally determined helical structure (flag fix_residues_to_).

### 6.8. *In vivo* transfection, staining and imaging

For the analysis of endogenous MuRF1 distribution in TA, its expression was induced by two weeks of N. ischiadicus denervation. Single myofibrils from TA were prepared as before [52], and endogenous MuRF1 was detected with three different polyclonal antibodies [21,37] (available from www.myomedix.com). Double labelling with desmin, coupled to AlexaFluor647, was used to determine Z-disc localized MuRF1 epitopes. All staining was done using standard protocols as previously described [53].

Expression of MuRF1^COS^ fused C-terminally to EGFP was by transfection of the expression vector into TA muscles, as previously described [54]. Ten days post-transfection, mice were anaesthetized, transfected muscles exposed and injected with the marker for acetylcholine receptors, α-bungarotoxin-AlexaFluor647, as previously described [37]. Mice were then transferred to a confocal microscope (DMRE TCS SP2, Leica Microsystems) and GFP fluorescence excited using a KrAr laser (488 nm). Emission was detected by a 63x/1.2NA HCX PL APO CS W CORR objective (Leica Microsystems) (immersion medium Visc-Ophtal gel, Winzer-Pharma) using 500–550 nm bandpass. Next, muscles were extracted and fixed in 4% PFA/PBS overnight at 4°C, washed in PBS for 30 min and embedded in 2% agarose. Longitudinal slices of 50 µm thickness were made using a Leica vibratome VT1000 S, permeabilized for 4–5 h in 0.1% Triton X-100 and washed in PBS. Sarcomeric actin was labelled with 250 nM phalloidin-TRITC (Life Technologies) in 2% BSA/PBS overnight; slices were then washed in 2% BSA/PBS for 1–2 h and embedded in Mowiol. GFP, phalloidin-TRITC and α-bungarotoxin-AlexaFluor647 were excited at 488, 561 and 633 nm, respectively. Emission was detected at 500–550, 570–620 and 650–750 nm bandpass.

## Supplementary Material

Figures S1-S6

## Data accessibility

Atomic coordinates and diffraction data have been deposited in the Protein Data Bank with accession code 4M3L.

## References

[1] Meroni G Diez-Roux G 2005 TRIM/RBCC, a novel class of ‘single protein RING finger’ E3 ubiquitin ligases. Bioessays 27, 1147–1157. (doi:10.1002/bies.20304) 16237670 10.1002/bies.20304

[2] Napolitano LM Meroni G 2012 TRIM family: Pleiotropy and diversification through homomultimer and heteromultimer formation. IUBMB Life 64, 64–71. (doi:10.1002/iub.580) 22131136 10.1002/iub.580

[3] Torok M Etkin LD 2001 Two B or not two B? Overview of the rapidly expanding B-box family of proteins. Differentiation 67, 63–71. (doi:10.1046/j.1432-0436.2001.067003063.x) 11428128 10.1046/j.1432-0436.2001.067003063.x

[4] Cambiaghi V Giuliani V Lombardi S Marinelli C Toffalorio F Pelicci PG 2012 TRIM proteins in cancer. Adv. Exp. Med. Biol. 770, 77–91. (doi:10.1007/978-1-4614-5398-7_6) 23631001 10.1007/978-1-4614-5398-7_6

[5] Cox TC 2012 The microtubule-associated C-I subfamily of TRIM proteins and the regulation of polarized cell responses. Adv. Exp. Med. Biol. 770, 105–118. (doi:10.1007/978-1-4614-5398-7_8) 23631003 10.1007/978-1-4614-5398-7_8

[6] Mayans O Labeit S 2012 MuRFs: specialized members of the TRIM/RBCC family with roles in the regulation of the trophic state of muscle and its metabolism. Adv. Exp. Med. Biol. 770, 119–129. (doi:10.1007/978-1-4614-5398-7_9) 23631004 10.1007/978-1-4614-5398-7_9

[7] Short KM Cox TC 2006 Subclassification of the RBCC/TRIM superfamily reveals a novel motif necessary for microtubule binding. J. Biol. Chem. 281, 8970–8980. (doi:10.1074/jbc.M512755200) 16434393 10.1074/jbc.M512755200

[8] Sardiello M Cairo S Fontanella B Ballabio A Meroni G 2008 Genomic analysis of the TRIM family reveals two groups of genes with distinct evolutionary properties. BMC Evol. Biol. 8, 225 (doi:10.1186/1471-2148-8-225) 18673550 10.1186/1471-2148-8-225 PMC2533329

[9] Reymond A 2001 The tripartite motif family identifies cell compartments. EMBO J. 20, 2140–2151. (doi:10.1093/emboj/20.9.2140) 11331580 10.1093/emboj/20.9.2140 PMC125245

[10] Woodsmith J Jenn RC Sanderson CM 2012 Systematic analysis of dimeric E3-RING interactions reveals increased combinatorial complexity in human ubiquitination networks. Mol. Cell Proteomics 11, M111 016162. 22493164 10.1074/mcp.M111.016162 PMC3394952

[11] Mrosek M Labeit D Witt S Heerklotz H von Castelmur E Labeit S Mayans O 2007 Molecular determinants for the recruitment of the ubiquitin-ligase MuRF-1 onto M-line titin. FASEB J. 21, 1383–1392. (doi:10.1096/fj.06-7644com) 17215480 10.1096/fj.06-7644com

[12] Centner T 2001 Identification of muscle specific ring finger proteins as potential regulators of the titin kinase domain. J. Mol. Biol. 306, 717–726. (doi:10.1006/jmbi.2001.4448) 11243782 10.1006/jmbi.2001.4448

[13] Pizon V Iakovenko A Van Der Ven PF Kelly R Fatu C Fürst DO Karsenti E Gautel M 2002 Transient association of titin and myosin with microtubules in nascent myofibrils directed by the MURF2 RING-finger protein. J. Cell Sci. 115, 4469–4482. (doi:10.1242/jcs.00131) 12414993 10.1242/jcs.00131

[14] Spencer JA Eliazer S Ilaria RL Jr Richardson JA Olson EN 2000 Regulation of microtubule dynamics and myogenic differentiation by MURF, a striated muscle RING-finger protein. J. Cell Biol. 150, 771–784. (doi:10.1083/jcb.150.4.771) 10953002 10.1083/jcb.150.4.771 PMC2175279

[15] Bodine SC 2001 Identification of ubiquitin ligases required for skeletal muscle atrophy. Science 294, 1704–1708. (doi:10.1126/science.1065874) 11679633 10.1126/science.1065874

[16] Wray CJ Mammen JM Hershko DD Hasselgren PO 2003 Sepsis upregulates the gene expression of multiple ubiquitin ligases in skeletal muscle. Int. J. Biochem. Cell Biol. 35, 698–705. (doi:10.1016/S1357-2725(02)00341-2) 12672461 10.1016/s1357-2725(02)00341-2

[17] Murton AJ Constantin D Greenhaff PL 2008 The involvement of the ubiquitin proteasome system in human skeletal muscle remodelling and atrophy. Biochim. Biophys. Acta 1782, 730–743. (doi:10.1016/j.bbadis.2008.10.011) 18992328 10.1016/j.bbadis.2008.10.011

[18] Clarke BA 2007 The E3 Ligase MuRF1 degrades myosin heavy chain protein in dexamethasone-treated skeletal muscle. Cell Metab. 6, 376–385. (doi:10.1016/j.cmet.2007.09.009) 17983583 10.1016/j.cmet.2007.09.009

[19] Cohen S Brault JJ Gygi SP Glass DJ Valenzuela DM Gartner C Latres E Goldberg AL 2009 During muscle atrophy, thick, but not thin, filament components are degraded by MuRF1-dependent ubiquitylation. J. Cell Biol. 185, 1083–1095. (doi:10.1083/jcb.200901052) 19506036 10.1083/jcb.200901052 PMC2711608

[20] Witt SH Granzier H Witt CC Labeit S 2005 MURF-1 and MURF-2 target a specific subset of myofibrillar proteins redundantly: towards understanding MURF-dependent muscle ubiquitination. J. Mol. Biol. 350, 713–722. (doi:10.1016/j.jmb.2005.05.021) 15967462 10.1016/j.jmb.2005.05.021

[21] Witt CC Witt SH Lerche S Labeit D Back W Labeit S 2008 Cooperative control of striated muscle mass and metabolism by MuRF1 and MuRF2. EMBO J. 27, 350–360. (doi:10.1038/sj.emboj.7601952) 18157088 10.1038/sj.emboj.7601952 PMC2168395

[22] McElhinny AS Kakinuma K Sorimachi H Labeit S Gregorio CC 2002 Muscle-specific RING finger-1 interacts with titin to regulate sarcomeric M-line and thick filament structure and may have nuclear functions via its interaction with glucocorticoid modulatory element binding protein-1. J. Cell Biol. 157, 125–136. (doi:10.1083/jcb.200108089) 11927605 10.1083/jcb.200108089 PMC2173255

[23] Eddins MJ Marblestone JG Suresh Kumar KG Leach CA Sterner DE Mattern MR Nicholson B 2011 Targeting the ubiquitin E3 ligase MuRF1 to inhibit muscle atrophy. Cell Biochem. Biophys. 60, 113–118. (doi:10.1007/s12013-011-9175-7) 21448668 10.1007/s12013-011-9175-7

[24] Moriscot AS Baptista IL Bogomolovas J Witt C Hirner S Granzier H Labeit S 2010 MuRF1 is a muscle fiber-type II associated factor and together with MuRF2 regulates type-II fiber trophicity and maintenance. J. Struct. Biol. 170, 344–353. (doi:10.1016/j.jsb.2010.02.001) 20149877 10.1016/j.jsb.2010.02.001 PMC2856802

[25] Perera S Mankoo B Gautel M 2012 Developmental regulation of MURF E3 ubiquitin ligases in skeletal muscle. J. Muscle Res. Cell Motil. 33, 107–122. (doi:10.1007/s10974-012-9288-7) 22426552 10.1007/s10974-012-9288-7 PMC3353113

[26] Fielitz J Kim MS Shelton JM Latif S Spencer JA Glass DJ Richardson JA Bassel-Duby R Olson EN 2007 Myosin accumulation and striated muscle myopathy result from the loss of muscle RING finger 1 and 3. J. Clin. Invest. 117, 2486–2495. (doi:10.1172/JCI32827) 17786241 10.1172/JCI32827 PMC1957544

[27] Parry DA Fraser RD Squire JM 2008 Fifty years of coiled-coils and α-helical bundles: a close relationship between sequence and structure. J. Struct. Biol. 163, 258–269. (doi:10.1016/j.jsb.2008.01.016) 18342539 10.1016/j.jsb.2008.01.016

[28] Walshaw J Woolfson DN 2001 Socket: a program for identifying and analysing coiled-coil motifs within protein structures. J. Mol. Biol. 307, 1427–1450. (doi:10.1006/jmbi.2001.4545) 11292353 10.1006/jmbi.2001.4545

[29] Mrosek M 2008 Structural analysis of B-Box 2 from MuRF1: identification of a novel self-association pattern in a RING-like fold. Biochemistry 47, 10 722–10 730. (doi:10.1021/bi800733z) 10.1021/bi800733z 18795805

[30] Seo J Cohen C 1993 Pitch diversity in α-helical coiled coils. Proteins 15, 223–234. (doi:10.1002/prot.340150302) 8456094 10.1002/prot.340150302

[31] Krissinel E Henrick K 2007 Inference of macromolecular assemblies from crystalline state. J. Mol. Biol. 372, 774–797. (doi:10.1016/j.jmb.2007.05.022) 17681537 10.1016/j.jmb.2007.05.022

[32] Strelkov SV Burkhard P 2002 Analysis of α-helical coiled coils with the program TWISTER reveals a structural mechanism for stutter compensation. J. Struct. Biol. 137, 54–64. (doi:10.1006/jsbi.2002.4454) 12064933 10.1006/jsbi.2002.4454

[33] Bibby J Keegan RM Mayans O Winn MD Rigden DJ 2012 AMPLE: a cluster-and-truncate approach to solve the crystal structures of small proteins using rapidly computed *ab initio* models. Acta Crystallogr. D 68, 1622–1631. (doi:10.1107/S0907444912039194) 23151627 10.1107/S0907444912039194

[34] Xu D Zhang Y 2012 *Ab initio* protein structure assembly using continuous structure fragments and optimized knowledge-based force field. Proteins 80, 1715–1735. 22411565 10.1002/prot.24065 PMC3370074

[35] Simons KT Kooperberg C Huang E Baker D 1997 Assembly of protein tertiary structures from fragments with similar local sequences using simulated annealing and Bayesian scoring functions. J. Mol. Biol. 268, 209–225. (doi:10.1006/jmbi.1997.0959) 9149153 10.1006/jmbi.1997.0959

[36] Le Rumeur E Hubert JF Winder SJ 2012 A new twist to coiled coil. FEBS Lett. 586, 2717–2722. (doi:10.1016/j.febslet.2012.05.004) 22584055 10.1016/j.febslet.2012.05.004

[37] Rudolf R 2012 Regulation of nicotinic acetylcholine receptor turnover by MuRF1 connects muscle activity to endo/lysosomal and atrophy pathways. Age (Dordr) 35, 1663–1674. (doi:10.1007/s11357-012-9468-9) 22956146 10.1007/s11357-012-9468-9 PMC3776120

[38] Chen SN Czernuszewicz G Tan Y Lombardi R Jin J Willerson JT Marian AJ 2012 Human molecular genetics and functional studies identify TRIM63, encoding muscle RING finger protein 1, as a novel gene for human hypertrophic cardiomyopathy. Circ. Res. 111, 907–919. (doi:10.1161/CIRCRESAHA.112.270207) 22821932 10.1161/CIRCRESAHA.112.270207 PMC3482312

[39] Kapinos LE Burkhard P Herrmann H Aebi U Strelkov SV 2011 Simultaneous formation of right- and left-handed anti-parallel coiled-coil interfaces by a coil2 fragment of human lamin A. J. Mol. Biol 408, 135–146. (doi:10.1016/j.jmb.2011.02.037) 21354179 10.1016/j.jmb.2011.02.037

[40] Garcia P Ucurum Z Bucher R Svergun DI Huber T Lustig A Konarev PV Marino M Mayans O 2006 Molecular insights into the self-assembly mechanism of dystrophia myotonica kinase. FASEB J. 20, 1142–1151. (doi:10.1096/fj.05-5262com) 16770013 10.1096/fj.05-5262com

[41] Elkins JM Amos A Niesen FH Pike AC Fedorov O Knapp S 2009 Structure of dystrophia myotonica protein kinase. Protein Sci. 18, 782–791. (doi:10.1002/pro.82) 19309729 10.1002/pro.82 PMC2762590

[42] Bell JL Malyukova A Holien JK Koach J Parker MW Kavallaris M Marshall GM Cheung BB 2012 TRIM16 acts as an E3 ubiquitin ligase and can heterodimerize with other TRIM family members. PLoS ONE 7, e37470 (doi:10.1371/journal.pone.0037470) 22629402 10.1371/journal.pone.0037470 PMC3357404

[43] Goujon M McWilliam H Li W Valentin F Squizzato S Paern J Lopez R 2012 A new bioinformatics analysis tools framework at EMBL-EBI. Nucleic Acids Res. 38, W695–699. (doi:10.1093/nar/gkq313) 20439314 10.1093/nar/gkq313 PMC2896090

[44] Cole C Barber JD Barton GJ 2008 The Jpred 3 secondary structure prediction server. Nucleic Acids Res. 36, W197–201. (doi:10.1093/nar/gkn238) 18463136 10.1093/nar/gkn238 PMC2447793

[45] Wolf E Kim PS Berger B 1997 MultiCoil: a program for predicting two- and three-stranded coiled coils. Protein Sci. 6, 1179–1189. (doi:10.1002/pro.5560060606) 9194178 10.1002/pro.5560060606 PMC2143730

[46] Kabsch W 2012 XDS. Acta Crystallogr. D 66, 125–132. (doi:10.1107/S0907444909047337) 20124692 10.1107/S0907444909047337 PMC2815665

[47] Rodriguez DD Grosse C Himmel S González C de Ilarduya IM Becker S Sheldrick GM Usón I 2009 Crystallographic *ab initio* protein structure solution below atomic resolution. Nat. Methods 6, 651–653. (doi:10.1038/nmeth.1365) 19684596 10.1038/nmeth.1365

[48] Emsley P Lohkamp B Scott WG Cowtan K 2010 Features and development of coot. Acta Crystallogr. D 66, 486–501. (doi:10.1107/S0907444910007493) 20383002 10.1107/S0907444910007493 PMC2852313

[49] Langer G Cohen SX Lamzin VS Perrakis A 2008 Automated macromolecular model building for X-ray crystallography using ARP/wARP version 7. Nat. Protoc. 3, 1171–1179. (doi:10.1038/nprot.2008.91) 18600222 10.1038/nprot.2008.91 PMC2582149

[50] Adams PD 2010 PHENIX: a comprehensive Python-based system for macromolecular structure solution. Acta Crystallogr. D 66, 213–221. (doi:10.1107/S0907444909052925) 20124702 10.1107/S0907444909052925 PMC2815670

[51] Ludtke SJ Baldwin PR Chiu W 1999 EMAN: semiautomated software for high-resolution single-particle reconstructions. J. Struct. Biol. 128, 82–97. (doi:10.1006/jsbi.1999.4174) 10600563 10.1006/jsbi.1999.4174

[52] Ojima K 2010 Dynamic distribution of muscle-specific calpain in mice has a key role in physical-stress adaptation and is impaired in muscular dystrophy. J. Clin. Invest. 120, 2672–2683. (doi:10.1172/JCI40658) 20592470 10.1172/JCI40658 PMC2912184

[53] Choi KR 2012 Rapsyn mediates subsynaptic anchoring of PKA type I and stabilisation of acetylcholine receptor *in vivo*. J. Cell Sci. 125, 714–723. (doi:10.1242/jcs.092361) 22331361 10.1242/jcs.092361

[54] Dona M Sandri M Rossini K Dell'Aica I Podhorska-Okolow M Carraro U 2003 Functional *in vivo* gene transfer into the myofibers of adult skeletal muscle. Biochem. Biophys. Res. Commun. 312, 1132–1138. (doi:10.1016/j.bbrc.2003.11.032) 14651990 10.1016/j.bbrc.2003.11.032

