# Supplementary material for: Molecular basis for the fold organization and sarcomeric targeting of the muscle atrogin MuRF1
Source: Open Biol. 2014 Mar 26;4(3):130172. doi: 10.1098/rsob.130172 (PMC3971405; doi:10.1098/rsob.130172)
Supplement: Figures S1-S6 [file rsob130172supp1.pdf]

# SUPPLEMENTARY MATERIALS

## Molecular basis for the fold organization and sarcomeric targeting of the muscle atrogin MuRF1

Barbara Franke[1], Alexander Gasch[2], Dayté Rodriguez[3], Mohamed Chami[4], Muzamil M. Khan[5], Rüdiger Rudolf[5,6], Jaclyn Bibby[1], Akira Hanashima[2], Julijus Bogomolovas[2], Eleonore von Castelmur[1], Daniel J. Rigden[1], Isabel Uson[3], Siegfried Labeit[2], Olga Mayans[1]*

**Figure S1: Knobs-into-hole packing of the crystallographic dimers of MuRF1-CC.** Residues are colored according to their position in the heptad-repeat as identified by the program SOCKET (1). Namely, *a* (red), *d* (green), *e* and *g* (blue) of the heptad repeats. The analysis indicated that in the AC dimer 18 residues exhibited a conventional CC packing (7,7,4 repeat), whilst the BD dimer had 25 residues in CC arrangement (7,7,7,4 repeat). This shows that the packing of chains in both palindromic arms exhibits a slight anisometry.

**Dimer B-D** **Dimer A-C**

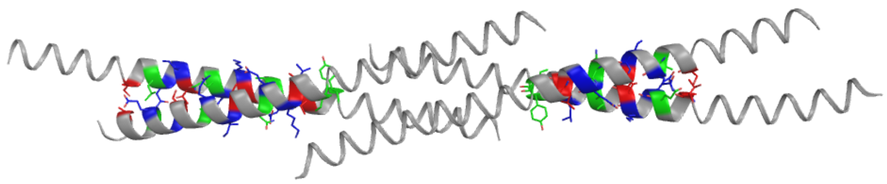

| Dimer B-D | Dimer A-C |
|---|---|
| SELLQRITQEQEEKLSFI | TQEQEEKLSFIEALIQQY |
| abcdefgabcdefgabcd | abcdefgabcdefgabcd |

**Figure S2:** ***Ab initio*** **models of the C-terminal fraction of the helical domain of MuRF1 (residues 214-327)**.

**a)** Top model generated using Quark (2); **b)** Superimposition of a family of 9 top models calculated in Quark; **c)** top model calculated in Rosetta (3), representative centroid of 150 models; **d)** Superimposition of Quark (rainbow) and Rosetta (grey) models. Despite a different identification of the C-terminus of the long helix $\alpha1$, the three-helical bundle topology of Quark and Rosetta models is strikingly similar.

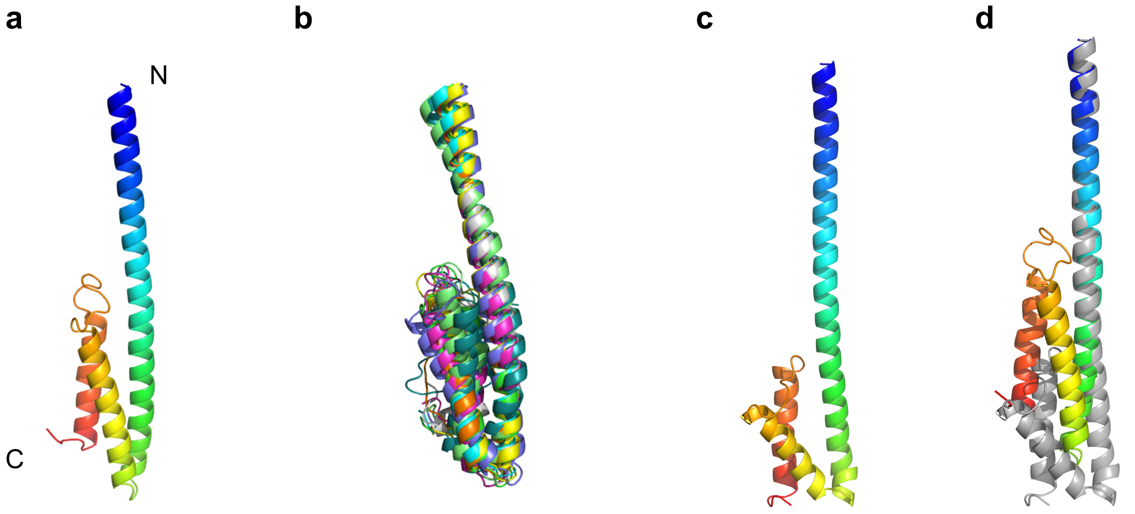

**Fig S3: SEC-MALLS measurements of MuRF1 component fractions**
The average molecular mass per volume unit (red) and the differential refractive index (blue) are shown. The theoretical mass of monomeric (continuous line) and dimeric (dashed line) states is indicated.

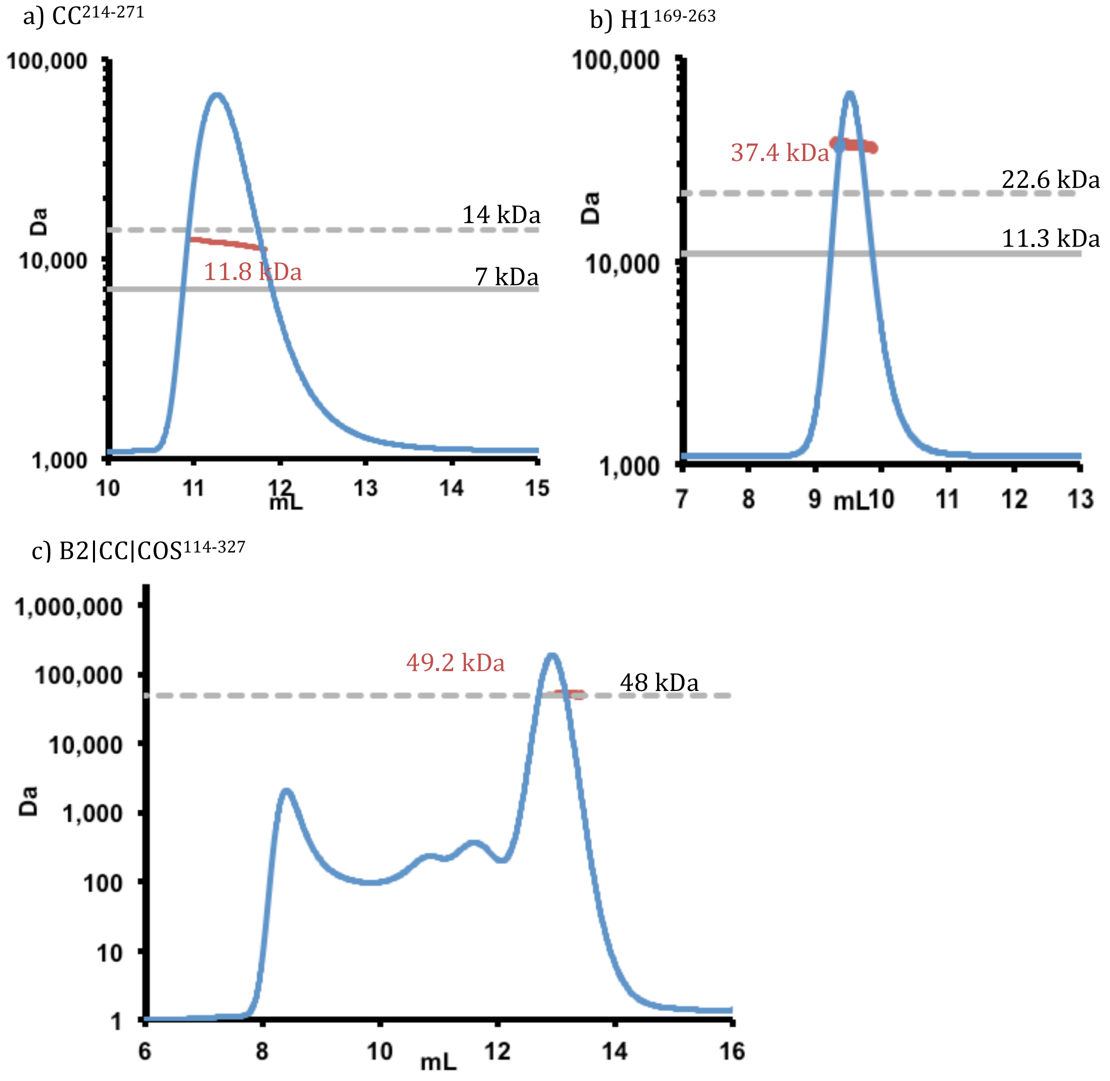


**Fig S4: Comparison of helix packing in the crystallographic structure of MuRF1$^{CC}$ (blue) and the Quark *ab initio* model that includes the COS-box (orange).** In the MuRF1$^{CC}$ crystallographic tetramer, helices arranged themselves along the same interfaces as those occupied by the COS-box α-hairpin in the *ab initio* models. This suggests that in the crystallographic assembly, amphipathic helices pack to best satisfy native interfaces.

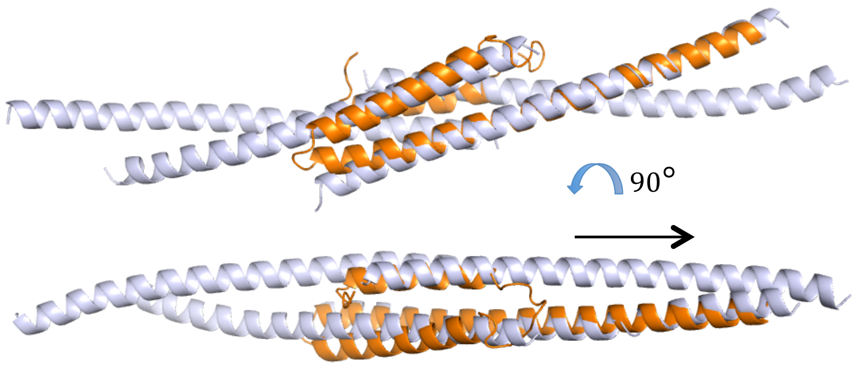

**Fig S5: Monitoring of MuRF1 H1 and COS-box complexation by gel filtration**

Individual and complexed samples were run on an analytical Superdex 200 10/300 GL column (GE Healthcare) in 20mM Tris pH 7.5, 200mM NaCl. The H1/COS complex was formed by mixing the components in a 1:1 ratio overnight at 4°C in the buffer above. **a.** Chromatograms. The amounts of sample injected onto the column were: $H1^{169\text{-}263}/COS^{261\text{-}330}$, 0.1μM of each component in 600μL; $H1^{169\text{-}263}$, 0.1μM in 175μL; $COS^{261\text{-}330}$, 0.1μM in 350μL. Fractions used in the accompanying SDS-PAGE gels are marked on the chromatogram (boxes). The gels (**b-d**) show equivalent fractions (collected every 250μL) from each run. In the presence of H1 (but not in isolation), the COS-box co-elutes at low volumes of exclusion.

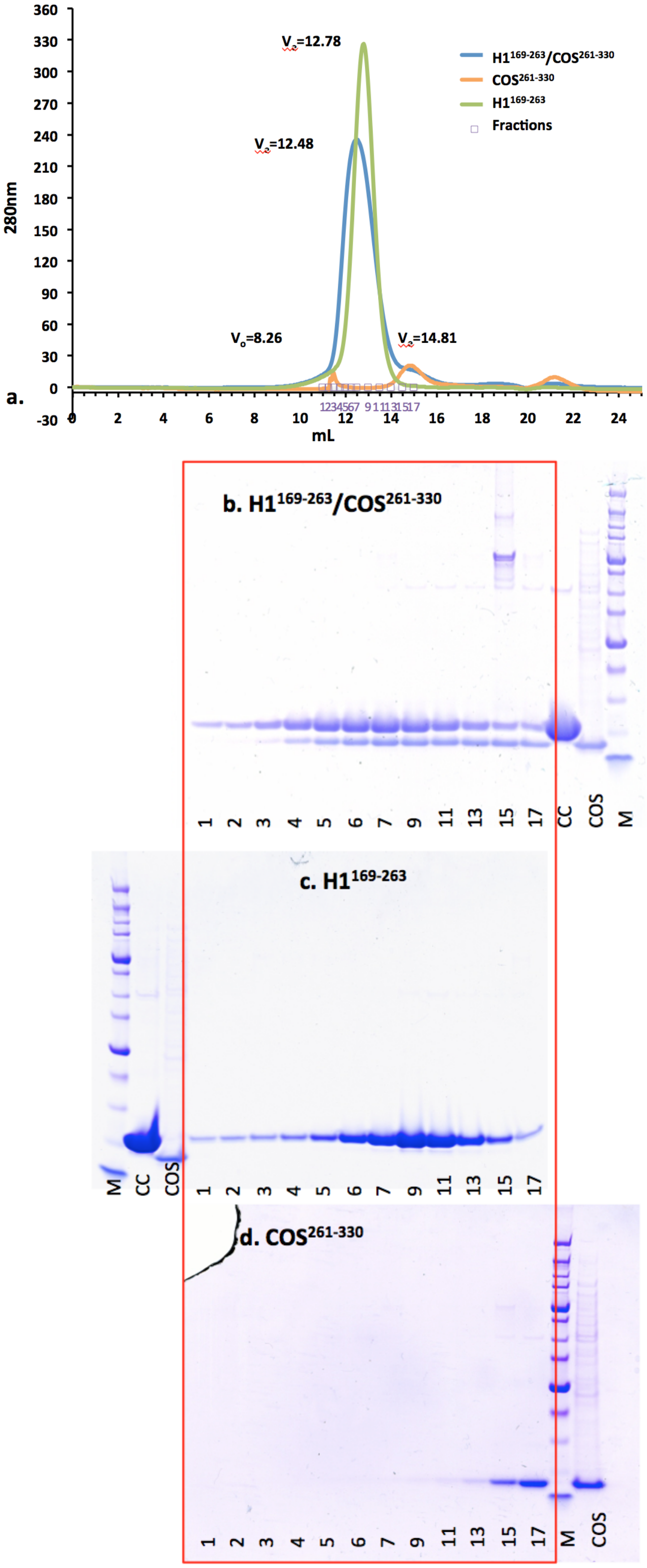

**Fig S6: GFP targeting in vivo**

In vivo targeting of transiently expressed GFP protein in transfected skeletal muscle. Depicted are confocal slices of neuromuscular junctions (A, pretzel-like structures), the corresponding GFP-expressing fibers (B), and the overlay of both (C). In the latter, neuromuscular junctions are in red and GFP signals in green. Arrowheads indicate AChR-positive puncta. Note that contrary to COS-GFP (Fig. 6d-g in main text), GFP does not exhibit any enrichment at the neuromuscular junctions or in sarcomeric striations.

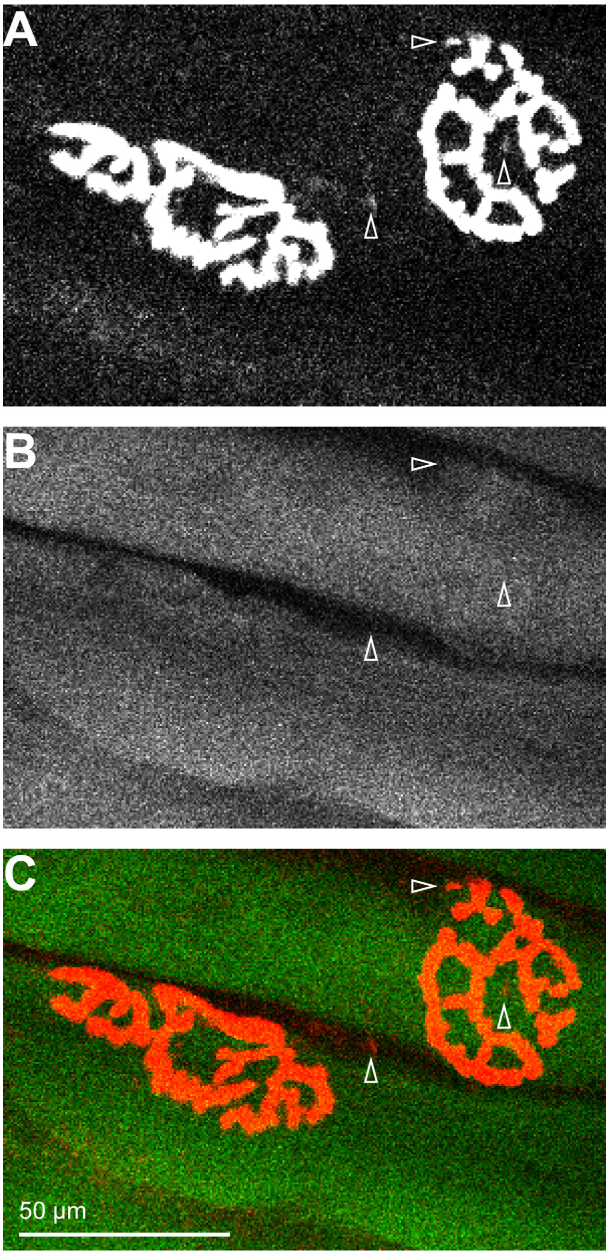


## References


1. Walshaw J, Woolfson DN (2001) Socket: a program for identifying and analysing coiled-coil motifs within protein structures. *J Mol Biol* 307: 1427-1450
2. Xu D, Zhang Y (2012) Ab initio protein structure assembly using continuous structure fragments and optimized knowledge-based force field. *Proteins* 80: 1715-1735.
3. Simons KT, Kooperberg C, Huang E, Baker D (1997) Assembly of protein tertiary structures from fragments with similar local sequences using simulated annealing and Bayesian scoring functions. *J Mol Biol* 268: 209-225.
